# Mediating effect of gestational weight gain on the preventive effect of exercise during pregnancy on macrosomia: a randomized clinical trial

**DOI:** 10.1186/s12884-024-06527-7

**Published:** 2024-05-22

**Authors:** Xuanjin Yang, Guifang Wang, Nana Liu, Yaxin Wang, Suhan Zhang, Hang Lin, Can Zhu, Li Liu, Yin Sun, Liangkun Ma

**Affiliations:** 1 National Clinical Research Center for Obstetric & Gynecologic Diseases, Department of Obstetrics and Gynecology, Peking Union Medical College Hospital, Chinese Academy of Medical Sciences & Peking Union Medical College, Dongcheng, Beijing, 100370 China; 2 Xingtai Xindu District Central Hospital, Hebei Xingtai, 054000 China; 3 Chinese Academy of Medical Sciences & Peking Union Medical College Nursing College, Beijing, 100144 China

**Keywords:** Exercise, Macrosomia, Gestational weight gain, Mediation analysis

## Abstract

**Objective:**

We sought to investigate the impact of individualized exercise guidance during pregnancy on the incidence of macrosomia and the mediating effect of gestational weight gain (GWG).

**Design:**

A prospective randomized clinical trial.

**Setting:**

A Hospital in Xingtai District, Hebei Province.

**Population:**

Older than 20 years of age, mid-pregnancy, and singleton pregnant women without contraindications to exercise during pregnancy.

**Methods:**

A randomized clinical trial was conducted from December 2021 to September 2022 to compare the effects of standard prenatal care with individualized exercise guidance on the incidence of macrosomia.

**Main outcome measure:**

Incidence of macrosomia.

**Results:**

In all, 312 singleton women were randomized into an intervention group (*N* = 162) or a control group (*N* = 150). Participants who received individualized exercise guidance had a significantly lower incidence of macrosomia (3.73% vs. 13.61%, *P* = 0.002) and infants large for gestational age (9.94% vs. 19.73%, *P* = 0.015). However, no differences were observed in the rate of preterm birth (1.86% vs. 3.40%, *P* = 0.397) or the average gestational age at birth (39.14 ± 1.51 vs. 38.69 ± 1.85, *P* = 0.258). Mediation analysis revealed that GWG mediated the effect of exercise on reducing the incidence of macrosomia.

**Conclusion:**

Individualized exercise guidance may be a preventive tool for macrosomia, and GWG mediates the effect of exercise on reducing the incidence of macrosomia. However, evidence does not show that exercise increases the rate of preterm birth or affects the average gestational age at birth.

**Trial registration:**

The trial is registered at www.clinicaltrails.gov [registration number: NCT05760768; registration date: 08/03/2023 (retrospectively registered)].

**Supplementary Information:**

The online version contains supplementary material available at 10.1186/s12884-024-06527-7.

## Recommendations

- 1. Pregnant women without contraindications should exercise according to their own conditions with management and supervision aided by information technology devices.
- 2. We should pay greater attention to the management of controllable and preventable factors, such as gestational weight gain, in patients under prenatal care.

## Introduction

Macrosomia refers to a neonatal birth weight greater than 4000 g [1]. The prevalence of macrosomia varies among different countries and regions, as developed countries have a prevalence rate of approximately 5% ~ 20%, while developing countries have a prevalence rate of approximately 0.5% ~ 14.9% [2]. In China, various studies have reported different rates of macrosomia, and a cross-sectional study including 14 provinces in China reported a rate of 7.83% in 2005 [3], while a multicentre study reported a rate of 7.3% in 2011 [4]. A nationally representative cross-sectional survey in mainland China illustrated that the overall prevalence of macrosomia in Chinese children younger than 6 years in 2013 was 7.35% [5]. The rate of macrosomia is consistently high and is associated with several maternal complications, such as caesarean delivery, postpartum haemorrhage, chorioamnionitis, soft birth canal laceration and even uterine and bladder rupture [6, 7]. In neonates, macrosomia increases the risk of shoulder dystocia, clavicle fractures, brachial plexus injury, respiratory distress, meconium aspiration syndrome, hypoglycaemia, polycythaemia and even mortality [1, 6–8]. In addition, macrosomia also affects the long-term health of offspring, as it increases the risk of overweight or obesity, hypertension, diabetes and cardiovascular disease [9]. Consequently, reducing the incidence of macrosomia is a public health issue that should be addressed.

Gestational weight gain (GWG) has been shown to be associated with macrosomia and large for gestational age (LGA) infants [10, 11]. Individual participant data from a meta-analysis revealed that excessive gestational weight gain (EGWG) was related to a greater risk of macrosomia (RR = 1.52, 95% CI: 1.33 ~ 1.73) than adequate GWG [12]. Furthermore, EGWG is regarded as an independent risk factor for macrosomia. An observational study revealed that for pregnant women with gestational diabetes mellitus (GDM), restriction of continued EGWG after commencing GDM management could reduce the risk of LGA infants [13]. Therefore, it is essential to control weight gain during pregnancy.

Exercise may be a useful method to control weight gain during pregnancy. A randomized controlled study revealed that pregnant women who did not exercise were 2.5 times more likely to give birth to newborns with macrosomia [14]. A systematic review and meta-analysis showed that exercise during pregnancy could decrease the incidence of macrosomia without affecting the likelihood of having an infant with preterm birth or low birth weight [15]. Additionally, exercise during pregnancy also plays an essential role in preventing other adverse pregnancy outcomes. Several systematic reviews and meta-analyses have shown that exercise during pregnancy can reduce the risk of EGWG, GDM, and hypertensive disorders, among others, during pregnancy [16, 17].

However, the mechanisms that underlie the effects of exercise on macrosomia are not fully understood. Therefore, we hypothesize that exercise during pregnancy reduces the incidence of macrosomia by decreasing GWG. In our study, we established an individualized exercise guidance programme for pregnant women and combined online and offline channels for monitoring. We aimed to explore the preventive effect of exercise on macrosomia and whether GWG has a mediating effect.

## Materials and methods

The RCT (NCT05760768) was a single-centre and parallel-group study conducted from December 2021 through September 2022 in Xingtai, Hebei Province. The research protocol was reviewed and approved by the Medical Ethics Committee of Xindu District Central Hospital in Xingtai City (reference number: 18). All participants provided written informed consent, and the ethics committee approved the consent procedure.

### Participants and randomization

**Fig. 1:**
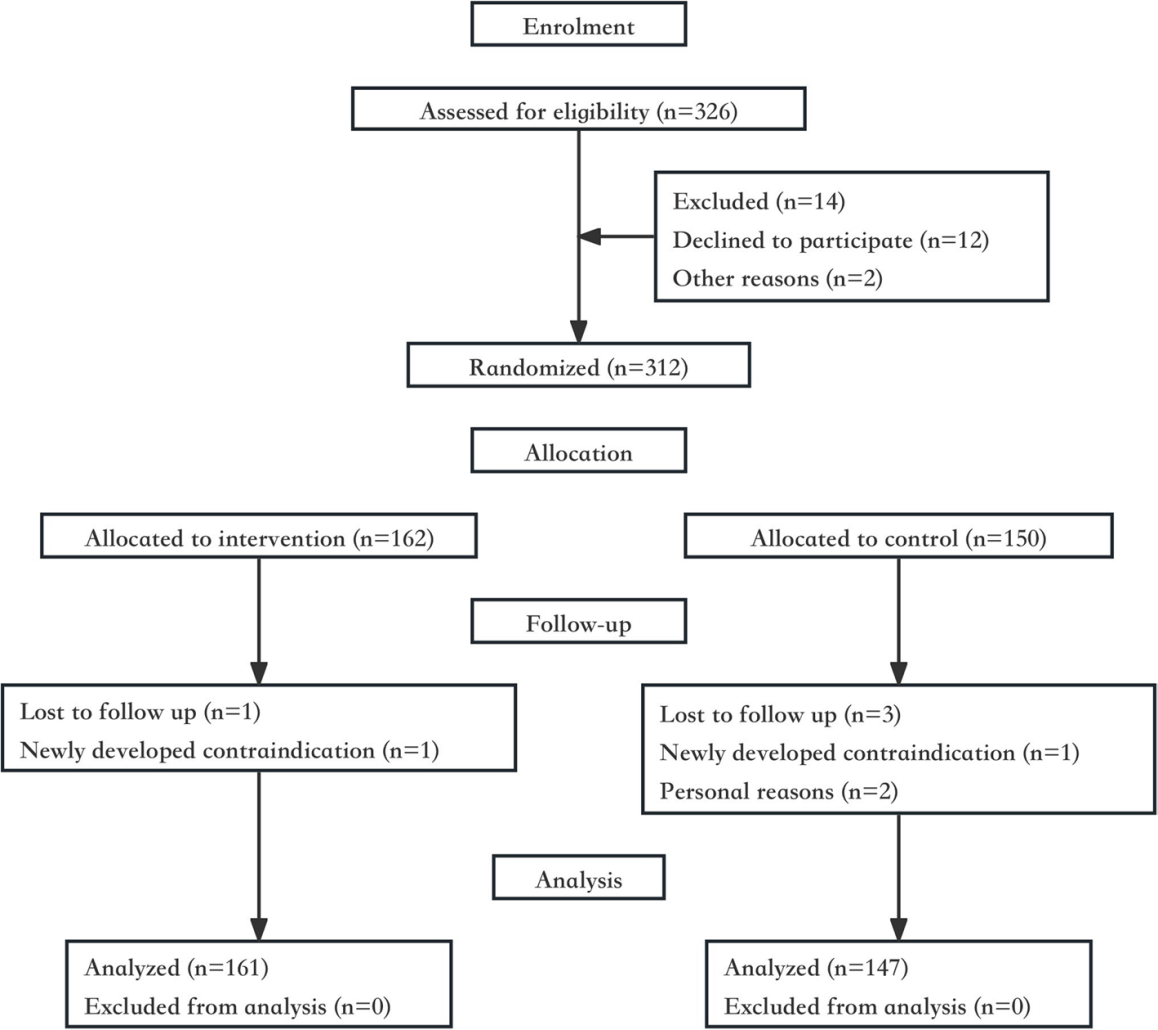
Trail profile

Overall, 326 pregnant women who visited Xingtai Xindu District Central Hospital for the first time between December 2021 and January 2022 were assessed for eligibility (Fig. 1). Singleton women aged older than 20 years who were in mid-pregnancy, who planned to undergo regular prenatal follow-ups and who gave birth at Xingtai Xindu District Central Hospital were included in this study. Pregnant women with contraindications to exercise during pregnancy, such as persistent vaginal bleeding or abdominal pain, cervical insufficiency, placenta previa, and severe heart failure, were excluded. Those who were unwilling to provide informed consent were also excluded.

In all, 312 pregnant women were randomly allocated to either an individualized exercise guidance (intervention) group or a standard prenatal care (control) group. The randomized participant assignment to the two groups alternatively followed the time of recruitment. The two researchers enrolled participants, generated the allocation sequence and assigned participants to interventions. Due to the nature of the intervention, all participants and research staff were aware of the allocations. The entire study was facilitated by obstetric specialists and qualified teachers who provided individualized exercise guidance. These teachers had received professional training in prenatal and postnatal exercise guidance.

### Participant demographics

Demographic and other information (pregravid weight and height), parity, medical and family history, and current pregnancy information were obtained at the time of enrolment. Information on prepregnancy exercise of the participants was collected through questionnaires. The ratio of weight to height squared was calculated to obtain the prepregnancy body mass index (BMI). According to weight monitoring and evaluation of pregnant Chinese women [18], prepregnancy BMI was classified into four categories: underweight (BMI < 18.5 kg/m^2^), normal weight (18.5 kg/m^2^ ≤ BMI < 24.0 kg/m^2^), overweight (24.0 kg/m^2^ ≤ BMI < 28.0 kg/m^2^) and obese (BMI ≥ 28.0 kg/m^2^).

### Standard prenatal care (control) group

Pregnant women randomly allocated to the control group received standard prenatal care, which was consistent with that of the intervention group. Participants in the control group also received general advice about the effects of physical activity and were not restricted from exercising on their own during pregnancy. No special dietary recommendations were given. A self-designed questionnaire was used to collect information on the exercise habits of the two groups, including exercise type, duration and frequency.

### Individualized exercise guidance (intervention) group

**Fig. 2:**
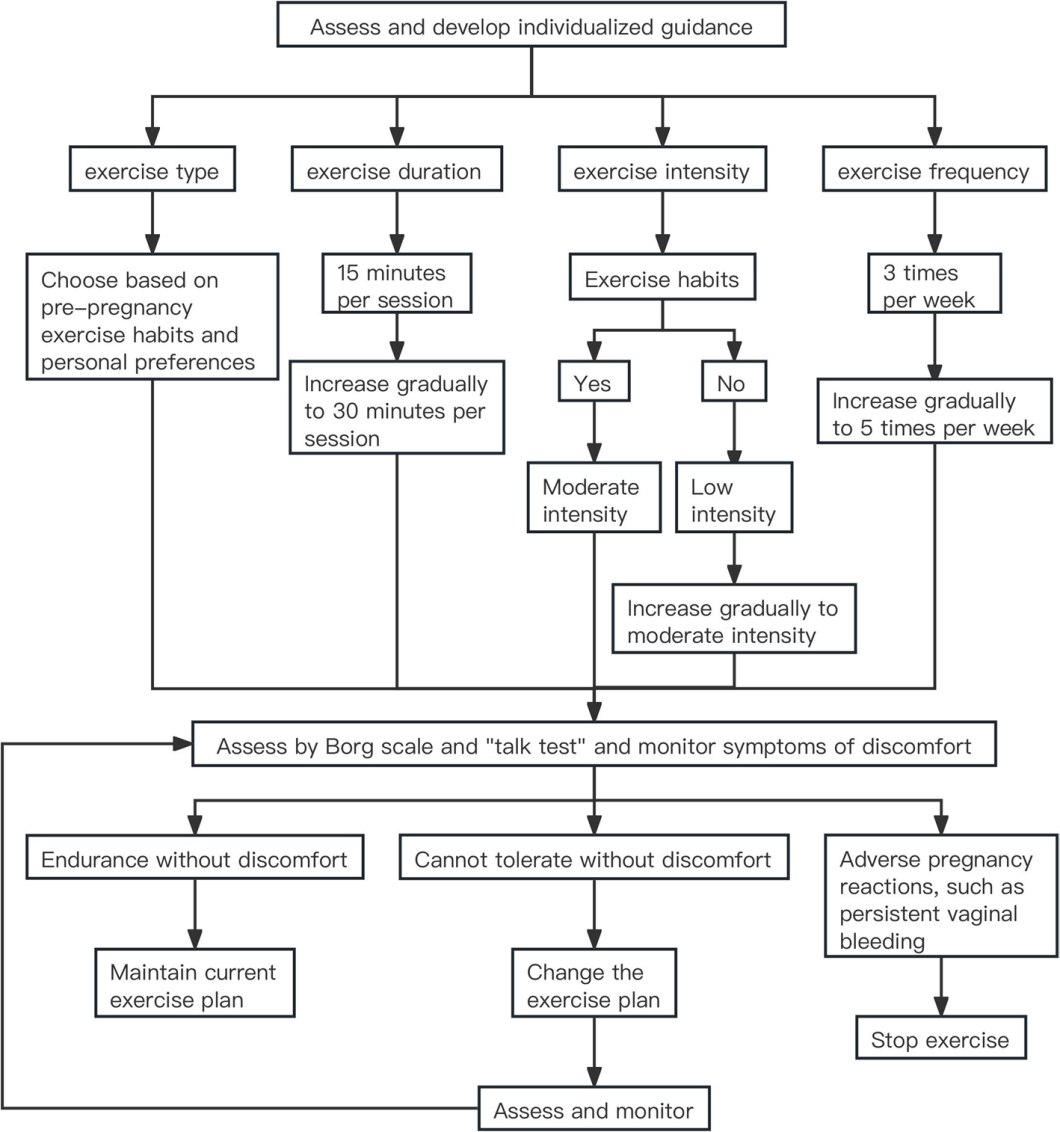
Individualized exercise guidance

Based on standard prenatal care, pregnant women randomly assigned to the intervention group received individualized exercise guidance immediately after randomization. The researchers informed the participants in the intervention group in detail about their contraindications to exercise and the timing of termination of exercise, which ensured patient safety during the intervention. According to age, prepregnancy BMI, previous physical activity habits, physical conditions and personal preferences, the researchers issued individualized exercise guidance, which was based on the American College of Obstetricians and Gynecologists (ACGO) committee recommendation [19] (Fig. 2). One of the exercise types was aerobic exercise, which includes jogging, fertility dance, swimming and yoga. The other was strength training, which was supplemented by tools such as elastic bands and dumbbells. The duration of exercise gradually increased from at least 15 min per session to at least 30 min per session. The exercise intensity was predominantly moderate. If a participant did not have a history of regular exercise before pregnancy, then a low-intensity exercise program that gradually increased to a moderate-intensity exercise program was recommended. The Borg Rating of Perceived Exertion (RPE) and the "talk test" were jointly used as indicators of exercise intensity. The Borg scale is the most commonly used method for assessing personal RPE and ranges from 6 to 20, representing exercise, which is considered “very, very light” to “very, very hard” [20]. For pregnant women, exercise intensity is generally controlled between Borg 13 and 14 [19, 21]. The "talk test" is another method: as long as a woman can have a conservation while exercising, she is likely not overexerting herself [22]. The frequency of exercise gradually increased from 3 times per week to 5 times per week. Each exercise included a warm-up session at the beginning and a stretching and relaxation session at the end. During the study, individualized exercise guidance was continuously adjusted by the researchers according to the participants’ tolerance and discomfort.

To guarantee quality, the researchers monitored and managed participants through both online and offline channels. Pregnant women in the intervention group had to ensure that they received individualized exercise guidance in a group setting as part of pregnancy education at the hospital at least three times per week and could exercise on their own at home for the remaining time. The researchers established a WeChat group to regularly send video, audio and text content to the participants in the intervention group and to collect information about the duration and type of exercise as well as discomfort felt during exercise through an electronic questionnaire. A personal data file was created for each participant and included information on exercise status and physical condition during pregnancy. The researchers summarized and provided feedback to all participants each week. Questions about pregnancy were collected once a week and were answered at the end of the week. Participants were encouraged to share their exercise or pregnancy experience and feelings in the WeChat group, which aimed to enhance peer support and peer education.

## Outcomes

### Primary outcome

Macrosomia refers to neonatal weight greater than 4000 g [1]. The primary outcome was the number of macrosomia cases (percentage/incidence).

### Secondary outcomes

The secondary outcomes included the rates of low birth weight (LBW) (birth weight < 2500 g), large for gestational age (LGA) (birth weight > 90th percentile for gestational age) and small for gestational age (SGA) (birth weight < 10th percentile for gestational age).

Gestational weight gain (GWG) was calculated according to the weight before delivery minus the pregravid weight and was stratified by prepregnancy BMI categories based on weight monitoring and evaluation of pregnant Chinese women [18]. Adequate gestational weight gain was as follows: 11 ~ 16 kg for underweight, 8 ~ 14 kg for normal weight, 7 ~ 11 kg for overweight, and 5 ~ 9 kg for obese. Lower weights than the above criteria are considered inadequate gestational weight gain, while higher weights are considered excessive gestational weight gain. The diagnoses of GDM, gestational hypertension and preterm birth obtained from the medical records were also included.

Postpartum weight retention (PPWR) was measured as the weight at 42 days after delivery minus pregravid weight. PPWR ≥ 5 kg was considered substantial and < 5 kg was considered insubstantial [23]. Diastasis recti abdominis (DRA) is defined as a palpated separation of ≥ 2 fingerbreadths either 4.5 cm above or 4.5 cm below the umbilicus. DRA was also measured at 42 days after delivery and was classified into three categories depending on the largest measured inter-rectus distance among the three locations [24]: 1) mild: 2–3 fingerbreadths; 2) moderate: 3–4 fingerbreadths; and 3) severe: 4 or more fingerbreadths.

### Statistical analysis

Power calculations for the primary outcome (the incidence of macrosomia) revealed a prevalence of 0.95% in the exercise group and 6.88% in the standard of care group according to a previous study [25]. Under these assumptions with a 2-sample comparison, a 10% level of significance and a power of 0.80, assuming a maximum loss to follow-up of 10%, approximately 150 pregnant women were needed for each group at baseline.

Continuous variables are presented as the means ± SDs, and categorical variables are presented as numbers and percentages. Differences in the means between two groups were evaluated using independent sample t tests and analysis of variance. Pearson’s X^2^ test was used for categorical variables. Missing data have been deleted. The mediation effect analysis was based on the following strategy proposed by Baron [26]: 1) the independent variable (IV) significantly affects the mediation factor; 2) in the absence of the mediation factor, the IV significantly affects the dependent variable (DV); 3) the mediation factor has a significant effect on the DV; and 4) the effect of the IV on the DV weakens upon the addition of a mediation factor in the model. A mediation model suitable for combining categorical and continuous variables was developed by Iacobucci [27]. The formulas are as follows:

If the mediation factor and dependent variable are continuous variables, the original formula of the Sobel test is applicable:

$${\text{Z}}=\frac{a\times b}{\sqrt{{b}^{2}{{s}_{a}}^{2}+{a}^{2}{{s}_{b}}^{2}}}$$

If the mediation factor or dependent variables are categorical variables, then the original formula of the Sobel test is rederived into a new formula:

$${\text{Z}}_{mediation}\left({\text{Z}}_m\right)=\frac{\frac a{s_a}\times\frac b{s_b}}{\sqrt{\left(\frac a{s_a}\right)^2+\left(\frac b{s_b}\right)^2+1}}$$

Here, a is the simple linear or logistic regression coefficient for the IV against the mediation factor, while b is the regression coefficient for the mediation factor against the DV in the binary linear or regression model. Additionally, Sa and Sb represent the standard deviations of a and b, respectively. Zm values exceeding |1.96|,|2.57|, and|3.90| (for the two-tailed test) are significant at a = 0.05, 0.01 and 0.001, respectively [28]. We conducted the statistical analyses using SPSS software (version 24.0; IBM Crop, Armonk, NY). The level of significance was set to < 0.05.

## Results

We concluded the study in September 2022 after all participants completed the follow-up. A total of 312 pregnant women met the inclusion criteria. After randomization, 1 pregnant woman in the intervention group was lost to follow-up because of newly developed contraindications, while 3 pregnant women in the control group were lost to follow-up because of newly developed contraindications (*n* = 1) and personal reasons (*n* = 2). In all, 308 pregnant women were analysed: 161 in the intervention group and 147 in the control group. Among them, data on PPWR and DRA were missing for 49 pregnant women. Pregnant women in the intervention group received regular individualized exercise guidance from enrolment until delivery.

### Maternal characteristics

Personal data, as shown in Table 1, were collected from all participants at the beginning of the study. The two groups were well matched at baseline, with no significant differences between them. After the study, significant differences were observed in the type, duration and frequency of exercise between the two groups (Supplementary Table 1). Only 42.86% (63/147) of the pregnant women in the control group engaged in exercise, which was predominantly walking, during pregnancy (Supplementary Fig. 1).

**Table 1:** Characteristics of the individualized exercise guidance (intervention) group and the standard prenatal care (control) group at study entry

	**intervention(*n* = 161)**	**control(*n* = 147)**	***P***
**Maternal characteristics**			
**Maternal age, y**	29.25 ± 4.09	29.99 ± 3.91	0.108
**Prepregnancy BMI, kg/m**^**2**^	23.32 ± 3.77	22.77 ± 3.76	0.199
**Gestational age, wk**	17.38 ± 2.74	16.87 ± 2.92	0.194
**Academic qualification categories, n%**			
Junior high school and below	22(13.66)	23(15.65)	0.314
High school	28(17.39)	37(25.17)	
College/university	107(66.46)	84(57.14)	
Postgraduate and above	4(2.48)	3(2.04)	
**Prepregnancy BMI categories, n%**			
Underweight	6(3.73)	10(6.80)	0.522
Normal weight	98(60.87)	92(62.59)	
Overweight	42(26.09)	31(21.09)	
Obese	15(9.32)	14(9.52)	
**Parity**			
0	88(54.66)	86(58.50)	0.497
1 +	73(45.34)	61(41.50)	
**Regular exercise before pregnancy**			
None	99(61.50)	104(70.75)	0.301
Walking	51(31.68)	38(25.85)	
Running	4(2.48)	2(1.36)	
Yoga	7(4.34)	3(2.04)	

### Macrosomia and other pregnancy outcomes

In the individualized exercise guidance group vs. the standard prenatal care group (Table 2), exercise significantly reduced the incidence of macrosomia (*P* = 0.002) and LGA (*P* = 0.015). No significant differences were found between the two groups in LBW or SGA, and no change was observed in gestational age at birth. Exercise reduced GWG (*P* < 0.001) and promoted adequate gestational weight gain (*P* < 0.001) in mothers in the intervention group compared with those in the control group. However, the two groups exhibited no significant differences in the incidences of GDM, gestational hypertension or preterm birth. Pregnant women randomized to the intervention group had a significantly lower PPWR (*p* < 0.001) than those in the control group. In addition, moderate and severe DRA were significantly less common in the intervention group than in the control group (*p* = 0.037).

**Table 2:** Effect of individualized exercise guidance on macrosomia and other pregnancy outcomes in all participants

	**intervention(*n* = 161)**	**control(*n* = 147)**	***P***
**Newborn**			
**Gestational age at birth, wk**	39.14 ± 1.51	38.69 ± 1.85	0.268
**Macrosomia, n%**			
Yes	6(3.73)	20(13.61)	0.002**
No	155(96.27)	127(86.39)	
**Low birth weight(LBW), n%**			
Yes	3(1.86)	5(3.40)	0.397
No	158(98.14)	142(96.60)	
**Large for gestational age(LGA), n%**			
Yes	16(9.94)	29(19.73)	0.015*
No	145(90.06)	118(80.27)	
**Small for gestational age (SGA), n%**			
Yes	7(4.35)	12(8.16)	0.164
No	154(95.65)	135(91.84)	
**Mother**			
**Gestational weight gain(GWG), kg**	11.52 ± 2.51	14.47 ± 4.64	0.000**
**Postpartum weight retention(PPWR), kg**	2.43 ± 4.14	7.01 ± 5.96	0.000**
**Gestational diabetes mellitus(GDM), n%**			
No	152(94.41)	141(95.92)	0.539
Yes	9(5.59)	6(4.08)	
**Gestational hypertension, n%**			
No	158(98.14)	145(98.64)	0.727
Yes	3(1.86)	2(1.36)	
**Preterm birth, n%**			
No	158(98.14)	142(96.60)	0.397
Yes	3(1.86)	5(3.40)	
**Evaluation of gestational weight gain, n%**			
Inadequate	8(4.97)	3(2.04)	0.000**
Adequate	111(68.94)	63(42.86)	
Excessive	42(26.09)	81(55.10)	
**Postpartum weight retention categories, n%**			
Insubstantial	89(63.12)	34(28.81)	0.000**
Substantial	52(36.88)	84(71.19)	
**Diastasis recti abdominis, n%**			
None	4(2.84)	1(0.85)	0.037*
Mild	84(59.57)	53(44.92)	
Moderate	48(34.04)	55(46.61)	
Severe	5(3.55)	9(7.63)	

*P* < 0.05, **P < 0.01

### Outcomes based on different prepregnancy BMIs

To investigate whether the effect of exercise on macrosomia was independent of prepregnancy BMI, we analysed the data by dividing the participants into two groups based on prepregnancy BMI (Table 3). A statistically significant difference was observed in the incidence of macrosomia between the intervention group and the control group in the underweight and normal weight pregnant women but not in the overweight and obese pregnant women. For mothers, exercise was effective in reducing GWG and PPWR and preventing substantial PPWR, irrespective of prepregnancy BMI.

**Table 3:** Effect of individualized exercise guidance on macrosomia and other pregnancy outcomes based on prepregnancy BMI

	**underweight and normal weight**			**overweight and obese**		
	**Intervention(*n* = 104)**	**Control (*n* = 102)**	***P***	**Intervention(*n* = 57)**	**Control(*n* = 45)**	***P***
**Newborn**						
**Macrosomia, n%**						
Yes	2(1.92)	12(11.76)	0.005**	4(7.02)	8(17.78)	0.094
No	102(98.08)	90(88.24)		53(92.98)	37(82.22)	
**Large for gestational age(LGA), n%**						
Yes	10(9.62)	19(18.63)	0.063	6(10.53)	10(22.22)	0.107
No	94(90.38)	83(81.37)		51(89.47)	35(77.78)	
**Mother**						
**Gestational weight gain(GWG), kg**	11.56 ± 2.26	14.90 ± 4.76	0.000**	11.46 ± 2.93	13.49 ± 4.24	0.008**
**Postpartum weight retention(PPWR), kg**	2.97 ± 4.12	8.16 ± 5.07	0.000**	1.55 ± 4.06	4.30 ± 7.05	0.022*
**Evaluation of gestational weight gain, n%**						
Inadequate	5(4.81)	2(1.96)	0.000**	3(5.26)	1(2.22)	0.191
Adequate	89(85.58)	52(50.98)		22(38.60)	11(24.44)	
Excessive	10(9.62)	48(47.06)		32(56.14)	33(73.33)	
**Postpartum weight retention categories, n%**						
Insubstantial	50(56.82)	19(22.89)	0.000**	39(73.58)	15(42.86)	0.004**
Substantial	38(43.18)	64(77.11)		14(26.42)	20(57.14)	
**Diastasis recti abdominis, n%**						
None	3(3.41)	0(0.00)	0.021*	1(1.89)	1(2.86)	0.410
Mild	51(57.95)	35(42.17)		33(62.26)	18(51.43)	
Moderate	31(35.23)	39(46.99)		17(32.08)	16(45.71)	
Severe	3(3.41)	9(10.84)		2(3.77)	0(0.00)	

*P* < 0.05, **P < 0.01

### Mediation analysis

**Fig. 3:**
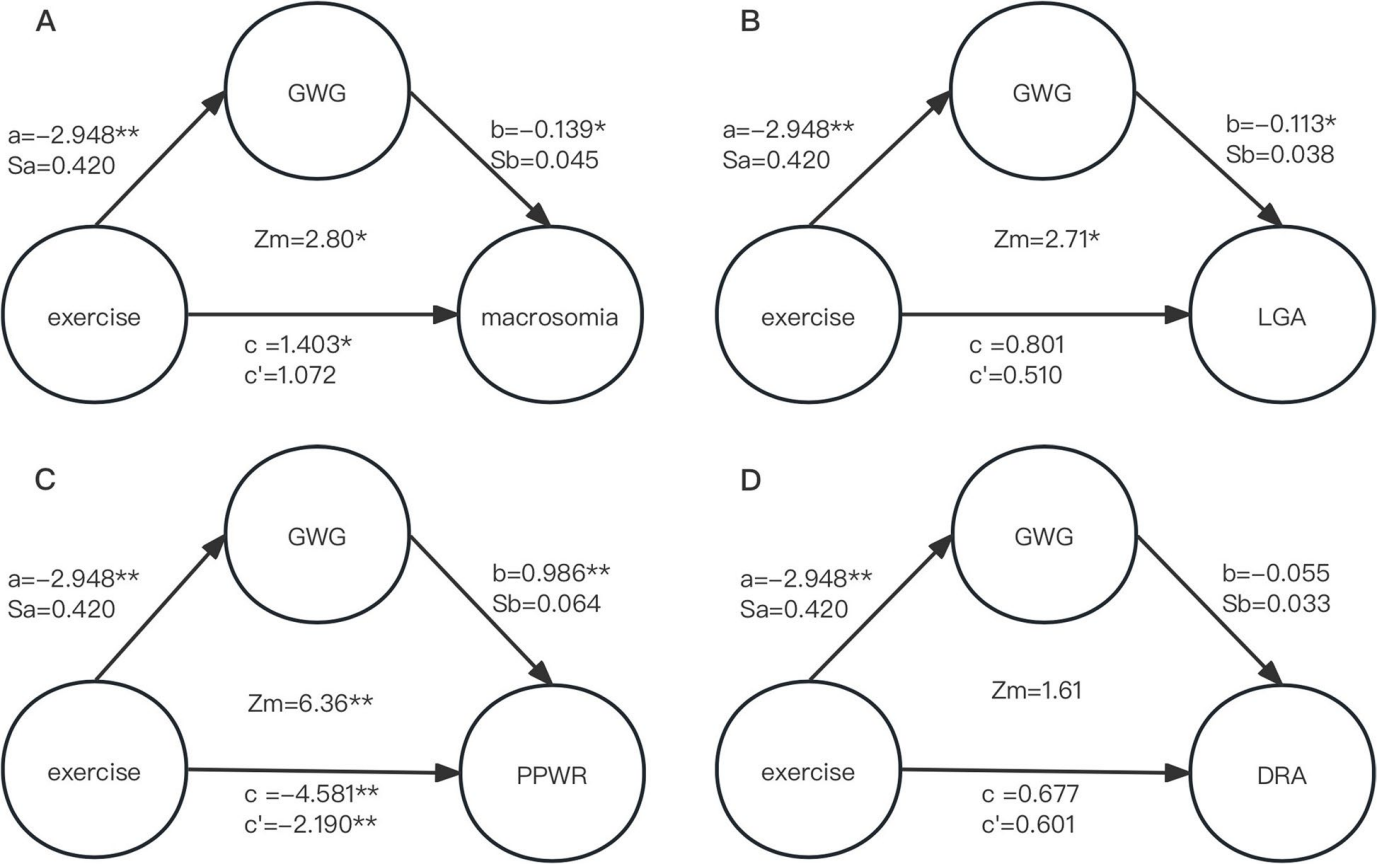
Mediating effect of GWG on exercise and **A** macrosomia, **B** LGA, **C** PPWR, and **D** DRA. **c** is the simple linear or logistic regression coefficient for the independent variable against the dependent variable in the absence of a mediating factor. **c**’ is the binary linear or logistic regression coefficient for the independent variable against the dependent variable in the presence of a mediating factor. a Simple linear or logistic regression coefficient for the independent variable against the mediating factor. **b** is the regression coefficient for the mediation factor against the dependent variable in the binary linear or regression model. Sa and Sb represent the standard deviations of **a** and **b**, respectively. Independent variable: exercise; mediation factor: GWG; dependent variables: macrosomia, LGA, PPWR and DRA. *P* < 0.05, **P < 0.01

Significant differences were found in GWG between the two groups, and the difference was also significant among the different prepregnancy BMI groups. Hence, we used GWG as a mediating factor to analyse its mediating effect between exercise during pregnancy and other outcomes (Fig. 3). Figure 3 A and B show that GWG mediated the effect of exercise during pregnancy and reduced the incidence of macrosomia (Zm = 2.80, *P* < 0.01) and LGA (Zm = 2.71, *P* < 0.01). Figure 3 C shows that GWG mediated the effect of exercise during pregnancy and decreased the PPWR (Zm = 6.36, *P* < 0.001). Figure 3 D illustrates that GWG did not mediate the effect of exercise during pregnancy on the prevention of moderate or severe DRA (Zm = 1.61, *P* > 0.05).

## Discussion

We conducted a prospective RCT to evaluate the efficacy of individualized exercise guidance for preventing macrosomia. Compared with previous studies, our study combined offline guidance and online remote management to improve the compliance of pregnant women. The main finding of our study was that GWG may play a mediating role in the preventive effect of exercise during pregnancy on macrosomia.

The overall rate of macrosomia in our study was 8.44%, with 13.61% in the standard prenatal care group and 3.73% in the individualized exercise guidance group. Our exercise intervention reduced the incidence of macrosomia, which was consistent with the conclusions of previous studies. A systemic review and meta-analysis also revealed that exercise during pregnancy was a protective factor against macrosomia [15]. Among overweight and obese pregnant women, although the occurrence of macrosomia was lower in the intervention group than in the control group, the difference was not statistically significant, which is consistent with the findings of Chen Wang et al. [29]. In our study, the small number of overweight and obese pregnant women may be the reason that no significant difference was found. However, regardless of the prepregnancy BMI, exercise during pregnancy reduced the incidence of macrosomia by approximately 10%, which agrees with the overall findings. Therefore, regardless of prepregnancy BMI, it is suggested that all pregnant women exercise appropriately during pregnancy according to their own conditions to decrease the occurrence of adverse pregnancy outcomes.

Our study also revealed that individualized exercise guidance could effectively reduce GWG regardless of prepregnancy BMI. A systematic review and meta-analysis revealed that pregnant women who performed physical activity during pregnancy gained 1.04 kg more than those who did not exercise [16], which was also confirmed in our study. In addition, our exercise intervention also prevented EGWG, which is regarded as a risk factor for childhood obesity [30] and adverse pregnancy outcomes, including GDM, hypertensive disorders of pregnancy, preterm birth, and caesarean delivery [31]. The incidence of EGWG in the standard prenatal care group was more than twice as high as that in the individualized exercise guidance group. However, after adjusting for prepregnancy BMI, the above findings did not hold true for overweight or obese pregnant women. Overweight and obesity are considered risk factors for excessive gestational weight gain [32], possibly because the preventive effect of exercise during pregnancy does not offset the harmful effects of overweight and obesity.

More importantly, we found that exercise during pregnancy reduces the occurrence of macrosomia by decreasing GWG; that is, GWG mediates the effect of exercise on macrosomia. Previous studies have predominantly focused on the protective effect of exercise on macrosomia, while our study also investigated the mediating role of GWG in this relationship. This result not only suggests that in prenatal care, attention should be given to the management of controllable and modifiable factors such as GWG to reduce prenatal complications and adverse pregnancy outcomes but also provides further insights into the mechanisms through which exercise influences foetal growth.

A variety of factors, such as genetic background, environmental factors, and maternal health status, predispose a newborn to macrosomia [1, 7, 33]. Multiple studies have confirmed that excessive gestational weight gain is a risk factor for macrosomia, regardless of race, region, and other factors [32, 34–36]. A cross-sectional, observational study revealed that even without GDM, the rate of GWG was a potentially modifiable contributor to insufficient β-cell function [37]. Another study revealed that more weight gain than what is recommended during pregnancy is associated with a greater decrease in insulin sensitivity between 15 and 35 weeks of gestation [38], which can lead to maternal and foetal hyperglycaemia. Consequently, the foetal release of insulin, insulin-like growth factors, and growth hormone can also increase, which, in turn, could lead to increased foetal fat deposition and larger foetal size [33].

Our study also revealed that exercise during pregnancy reduced the incidence of LGA infants and that GWG mediated this effect. Similarly, a systematic review and meta-analysis revealed that the likelihood of delivering LGA infants decreased by 17% among normal weight women who exercised during pregnancy [39]. Excessive gestational weight gain was found to be a risk factor for LGA infants, independent of the impact of prepregnancy BMI on LGA [32, 36, 40]. Thus, exercise during pregnancy reduces the occurrence of LGA by reducing GWG.

We also found that exercise during pregnancy may effectively reduce PPWR by decreasing GWG. Substantial postpartum weight retention increases the risk of subsequent hypertension and diabetes, as well as the risk of developing GDM and gestational hypertension in subsequent pregnancies [41]. Excessive gestational weight gain is one of several factors that influences PPWR [17]. However, we did not find that GWG mediated the effect on the prevention of moderate or severe DRA. We speculate that this may be due to exercise strengthening the abdominal wall muscles, thereby reducing the occurrence of moderate or severe DRA.

Furthermore, our study did not find that exercise during pregnancy increased adverse side effects. The preterm birth rate in the standard prenatal care group was 3.40%, while that in the individualized exercise guidance group was lower than that at only 1.86%. No statistically significant differences were observed in mean gestational age at birth between the two groups. A review revealed that exercise did not increase the risk of preterm birth [42]. A systematic review and meta-analysis also suggested that exercise did not affect preterm birth [16].

Our study showed that exercise during pregnancy is a protective factor against macrosomia, large for gestational age infants, and excessive gestational weight gain. Pregnancy is not an exclusion criterion for exercise. The ACOG [19] recommends that pregnant women without contraindications to exercise engage in moderate-intensity exercise for 20–30 min per day or at least 4 times per week. The SOCG and CSEP [43] also recommend that pregnant women without contraindications to exercise accumulate at least 150 min of moderate-intensity physical activity per week, combining aerobic and resistance exercise. However, each pregnant woman has different personal circumstances, and individualized exercise plans that are dynamically adjusted according to her physical changes during pregnancy need to be developed. It is also difficult to implement a pregnancy exercise plan entirely in the hospital. Therefore, it is recommended that all pregnant women without contraindications perform physical activity during pregnancy according to their own conditions with management and supervision aided by information technology devices. We also found that only a few pregnant women in the control group voluntarily exercised during pregnancy, which suggested that we should strengthen the education and management of exercise during pregnancy in perinatal care.

### Strengths and weaknesses

First, the major strength of our study is that it was an RCT with high adherence (> 90%) in the two groups, which ensures the reliability and authenticity of the study results. This high adherence may be related to the development of individualized exercise guidance for participants based on their personal situations. Second, our study combined offline exercise guidance and online remote supervision. Pregnant women randomized to the intervention group received individualized exercise guidance at least 3 times per week in the hospital and exercised on their own at home for the remainder of their pregnancy. Moreover, the researchers established a WeChat group to understand the exercise situation of each participant and to strengthen their knowledge, education and peer education. Third, our study not only reaffirmed that exercise during pregnancy reduces the incidence of macrosomia but also revealed that GWG mediates this effect. Our study also highlights that interventions that focus on reducing modifiable risk factors, such as GWG, should be incorporated into prenatal care.

Our study also has some limitations. First, our study did not consider the impact of dietary patterns of energy intake during pregnancy on outcomes. Although both groups received standard prenatal care, including dietary advice during pregnancy, personal compliance may vary. In future research, both exercise and diet together should be considered. Furthermore, our study did not consider the intensity of daily physical activity, such as work and housework.

Further research is needed to investigate whether overall lifestyle interventions during pregnancy, including exercise, diet and other factors, can more effectively improve pregnancy outcomes. At the same time, it is essential to perform follow-up to explore the incidence of overweight/obesity and other cardiovascular diseases in the offspring of pregnant women who exercise during pregnancy.

### Supplementary Information

**Supplementary Material 1.**

## Data Availability

The data and materials that support the findings of this study are available from the corresponding author upon reasonable request.
