# Supplementary material for: Mediating effect of gestational weight gain on the preventive effect of exercise during pregnancy on macrosomia: a randomized clinical trial
Source: BMC Pregnancy Childbirth. 2024 May 22;24:384. doi: 10.1186/s12884-024-06527-7 (PMC11112785; doi:10.1186/s12884-024-06527-7)
Supplement: Supplementary file 1 — Supplementary Material 1. [file 12884_2024_6527_MOESM1_ESM.docx]

**Supplementary Materials**

**Supplementary Figure1**

**Exercise types of the individualized exercise guidance (intervention) group and standard prenatal care (control) group during pregnancy**

1. Intervention group; (B) Control group.

**Supplementary Table 1**

**Exercise situation of the individualized exercise guidance (intervention) group and standard prenatal care (control) group during pregnancy**

|  | **Intervention (n=161)** | **Control (n=147)** | **P** |
| --- | --- | --- | --- |
| **Frequency, times per week** | 4.12±1.57 | 1.37±1.68 | 0.000*** |
| 0 | 0 (0) | 54 (36.74) | 0.000*** |
| 1 or 2 | 32 (19.88) | 73 (49.66) |  |
| 3 or 4 | 57 (35.40) | 8 (5.44) |  |
| ≥5 | 72 (44.72) | 12 (8.16) |  |
| **Duration, min** | 35.59±6.95 | 17.04±14.96 | 0.000*** |
| 0 | 0 (0) | 54 (36.73) |  |
| >0 and <30 | 20 (12.42) | 53 (36.05) | 0.000*** |
| ≥30 | 141 (87.58) | 40 (27.22) |  |
| **Cumulative weekly time, min** | 148.85±62.68 | 36.26±46.22 | 0.000*** |
| <150 | 72 (44.72) | 139 (94.56) | 0.000*** |
| ≥150 | 89 (55.28) | 8 (5.44) |  |

***P<0.001
